# Specific autonomy recovery programme in a comprehensive rehabilitation on functionality and respiratory parameters in oncological patients with dyspnoea. Study protocol

**DOI:** 10.1186/s12912-021-00633-z

**Published:** 2021-07-05

**Authors:** Eduardo José Fernández-Rodríguez, Jesús González-Sánchez, Ana Silvia Puente-González, José Ignacio Recio-Rodríguez, Celia Sánchez-Gómez, Roberto Méndez-Sánchez, Juan Jesús Cruz-Hernández, María Isabel Rihuete-Galve

**Affiliations:** 1 Department of Nursing and Physiotherapy, University of Salamanca, Salamanca, Spain; 2 Medical Oncology Service, University Hospital Complex of Salamanca, Salamanca, Spain; 3 Institute of Biomedical Research of Salamanca (IBSAL), Salamanca, Spain; 4 Department of Developmental and Educational Psychology, University of Salamanca, Salamanca, Spain; 5 Department of Medicine, University of Salamanca, Salamanca, Spain

**Keywords:** Cancer, Oncology, Dyspnoea, Nursing, Comprehensive rehabilitation, Autonomy, Exercise, Functionality, Quality of live

## Abstract

**Background:**

Survival in cancer patients has increased exponentially in recent years, with multiple side effects caused by treatments. Cancer-related asthenia and dyspnea are among them, which represent a serious health problem, with considerable limitations and reduced quality of life. An implementation of the conventional clinical practice, developed through physical exercise, may be useful in controlling dyspnoea. This study aims to compare the effects of a comprehensive rehabilitation implementing a programme of multimodal physical exercise with a specific autonomy recovery programme, versus an isolated intervention using the physical exercise programme alone, on the functionality, physical performance and respiratory parameters in oncologycal patients with dyspnea.

**Methods:**

This is a protocol por an experimental, prospective, randomized, parallel-controlled clinical trial, with two arms design of fixed assignment with an experimental and control groups. It will conduct in the Oncology Hospitalisation Unit at the University Hospital Complex of Salamanca, using consecutive sampling to select 50 participants with oncological dyspnoea who are hospitalised at the time of inclusion. After baseline assessment, participants will be randomised into the groups. Experimental group will complete Comprehensive Rehabilitation with the autonomy recovery and the multimodal exercise programmes, and in the control group, only the multimodal exercise programme will be carried out. The primary outcomes will be basic activities of daily living (Barthel Index) and degree of dyspnoea (MRC scale). Additionally, physical performance will be evaluated with the Short Physical Performance Battery (SPPB), as will the oxygen saturation in the blood using pulse oximetry, fear/avoidance of movement with the Tampa Scale of Kinesiophobia (TSK), and the quality of life of the oncology patient (ECOG performance scale).

**Discussion:**

The results of this study may be translated to clinical practice, incorporating a specific autonomy recovery programme into comprehensive rehabilitation programmes of care for cancer patients with dyspnoea. Increase in the survival of patients with cancer includes multiple side effects as cancer-related asthenia and dyspnea, which represents a serious health problem. The current study addresses to improve the conventional clinical practice by proposing an integral, rehabilitative approach, to implement education and training for oncology patients with dyspnea to increase their quality of life.

**Trial registration:**

ClinicalTrials.gov; ID: NCT04766593. (February 23, 2021).

## Background

In recent years, the improvement in cancer treatments and broad support for preventive strategies have enabled us to reach a higher rate of early diagnosis and a better understanding of the oncologic pathology itself, thus achieving an exponential increase in the five-year survival of patients with cancer [1].

Linked to that increase in survival, and due to the resulting increase in the lines of treatment employed, we are seeing greater side effects, which have repercussions in aspects such as the patients’ functionality and their quality of life [2]. Some of these side effects include cancer-related asthenia, anxiety, and dyspnoea [3]. Dyspnoea may represent a serious health problem, with considerable limitations for the individuals. In some patients with advanced-stage cancer, this dyspnoea may represent a clinical sign of the final phase of the disease [4]. Approximately 41% of the patients in palliative care have dyspnoea, and 46% of those describe it as being moderate or severe [5, 6].

The majority of patients perceive this dyspnoea to be a limiting factor that is out of their control, which leads them to adopt behaviours which have negative repercussions on their functionality [7], such as cancer patients creating patterns of fear/avoidance of movement or of physical activity, as is often the case also with patients with chronic pain, chronic fatigue syndrome, or fibromyalgia [8, 9]. It is similar to when patients with respiratory problems decrease their level of activity to adapt to their symptomatology. This favours a worsening of their physical state and of the dyspnoea upon exertion, known as the “cycle of the respiratory patient” [10].

To control the dyspnoea, we believe that the measures employed in conventional clinical practice, which have been mainly developed through physical exercise, may be implemented. Reeducation and work on the activities of daily living, together with a programme of physical exercise, as part of an integral approach of functional reeducation, may be a good intervention for dyspnoea in oncology patients [11]. For this reason, in this clinical trial we are proposing an intervention with an integral perspective that is based on a rehabilitative approach, with the objective of integrating education and training of the oncology patient with dyspnoea in the adaptation of their day-to-day activities while their cardiopulmonary reserves are gravely reduced, with the aim of recovering their capacity to carry out their occupational roles.

After analysing previous studies, we have observed that the most-employed intervention is physical exercise, which has been shown to give good results in the majority of dyspnoea cases [12, 13]. Additionally, it appears that multimodal physical exercise programmes, which combine different types of exercise, such as aerobic exercise and strength training primarily, appear to have better overall results in cancer patients [14–16]. However, there are not many studies which correlate whether this improvement in symptoms has had positive repercussions on the patients’ functionality when carrying out their activities of daily living. We believe that, in addition to employing physical exercise to improve the patients symptomatology, this intervention should be geared towards increasing the individuals autonomy, and that this will make it possible to generalise in their daily life the symptomatic clinical effects obtained through the therapeutic process, which will be inexorably linked to an improvement in their quality of life.

As such, we are proposing as an intervention an Oncological Functional Reeducation Programme, to be used in addition to the intervention with physical exercise. It would be a reeducation in the completion of the activities of daily living, so as to foment the recovery of personal autonomy, and thus to observe to what degree this improves the results in clinical practice. The findings will provide important insight into the development of an effective care model with a comprehensive rehabilitation programme that includes a specific autonomy recovery programme in oncological patients with dyspnoea.

## Methods/design

### Aims

In accordance with that laid out above, the primary aim of this study is to contrast our hypothesis, that the intervention using a specific programme for the recovery of autonomy by means of reeducation on the activities of daily life, when added to multimodal physical exercise, will increase the beneficial effects of physical exercise as an isolated intervention on the functionality, physical performance, and respiratory parameters in oncology patients with dyspnea. The objectives seek to compare the effects produced by the implementation of a programme of multimodal physical exercise and a specific programme for recovering autonomy, with an isolated intervention using physical exercise, on the functionality and physical performance of cancer patients with dyspnoea.

### Design

This study is an experimental, prospective, randomized, parallel-controlled clinical trial, with two arms design of fixed assignment with an experimental and a control groups. It will conduct during a year in the Oncology Hospitalisation Unit at the University Hospital Complex of Salamanca (CAUSA), using consecutive sampling to select the participants with oncological dyspnoea who are hospitalised at the time of inclusion.

### Sample/participants

#### Participants

The patients will be recruited from the Oncology Hospitalisation Unit at the CAUSA, and must meet the following selection criteria: A) Inclusion criteria: Participants must have a pathological diagnosis of an oncological disease, be over 18 years of age, be hospitalised at the time of recruitment in the Oncology Unit at CAUSA, meet dyspnoea parameters of 2 or more points on the Medical Research Council scale (MRC), receive fewer than 85 points on the Barthel Index, and have signed an informed consent form indicating voluntary agreement to participate in the study. B) Exclusion criteria: not having an adequate cognitive state to be able to comprehend and follow the orders provided (fewer than 23 points on the Mini Mental State Examination, MMSE), having a haemoglobin level lower than 10 g/dL, being an active smoker at the time of recruitment. C) Withdrawal criteria: disease progression which brings the patient to a terminal situation or to death, and non-completion of the follow-up and final assessments.

#### Sample size

The sample size has been estimated based on the potential modification of the most stringent of the principal variables of the study—the score on the Barthel Index. For this, as a reference, we have used the results obtained in a study with similar characteristics, in which the scoring on the Barthel Index was modified by 7.5 points [17]. With these premises, accepting an alpha risk of 0.05 and a beta risk of 0.2 in a bilateral contrast, we will need 25 subjects in the first group and 25 subjects in the second group in order to detect a difference greater than or equal to 7.5 units. The assumed common standard deviation is 8.3. We estimate a rate of 20% lost to follow-up. The sample size was estimated using the programme EPIDAT 4.2.

#### Sample assignment

**Fig. 1:**
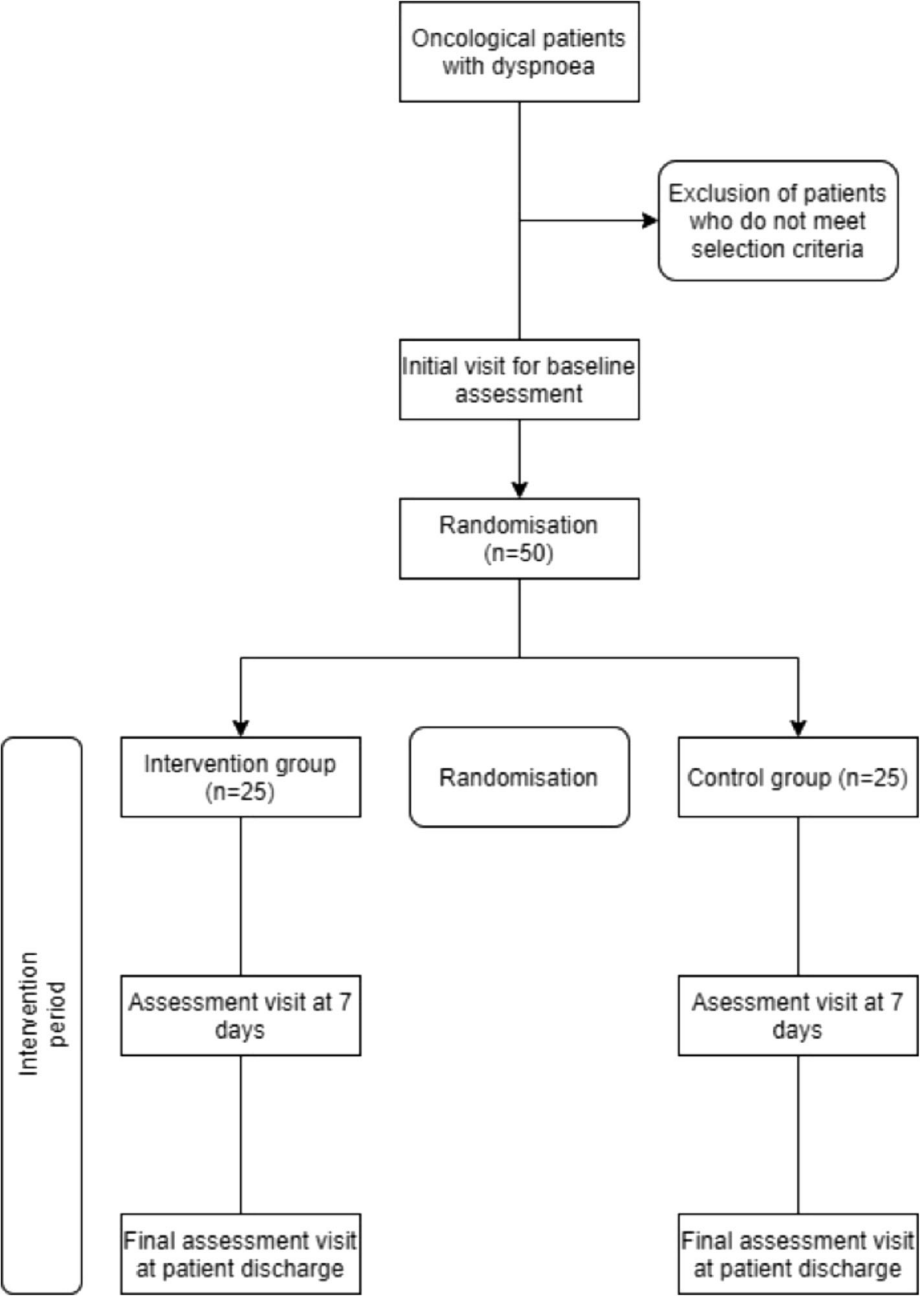
Flow Chart of the study. The enrolled participants are to be randomly assigned to one of the study conditions, with assessments to be taken at three pre-specified time points

After meeting the selection criteria listed above and being recruited for the study, the individuals will be assigned via a simple randomisation process to one of the two conditions of the study: Experimental Condition or Control Condition. As detailed in Fig. 1.

#### Randomisation

We will employ a simple randomisation process, which consists of generating a table of random numbers that is the same size as the estimated sample in the study. This will be done using Microsoft Excel 2020. Each of the randomised numbers will be assigned a condition: for even numbers, the subject will be assigned to the Experimental condition, and for odd numbers, they will be assigned to the Control condition. The work of the randomisation sequencing, the recruitment of participants, and of assigning them to a group will be completed by research personnel who are not related to the assessments nor to the implementation of the intervention, which will prevent potential biases in the study.

#### Blinding

Given the nature of the intervention, the participating subjects will not be blinded. However, to minimise any contamination between groups, the investigator responsible for the measurements of the study at each assessment and for the statistical analysis will be blinded, thus increasing the scientific quality in the study process.

### Procedures and data collection

#### Evaluations and study plan

Three assessments will be completed in this study: one at the beginning, another after 7 days, and a final one before the patient is discharged from hospital. After recruitment and before randomisation and assignment to the corresponding group, all individuals will complete a baseline assessment (BA), which includes recording the independent variables and the variables of the primary and secondary results of the study. Then, while the patients are still in hospital, the subjects will be randomly assigned to the control or the experimental group, with the corresponding intervention applied in each of them. Later, two more assessments will be completed, one after 7 days, considered to be the follow-up assessment (FUA), and another at the time of discharge from the hospital, considered to be the final assessment (FA). This can be observed in more detail in Table 1.

**Table 1:**
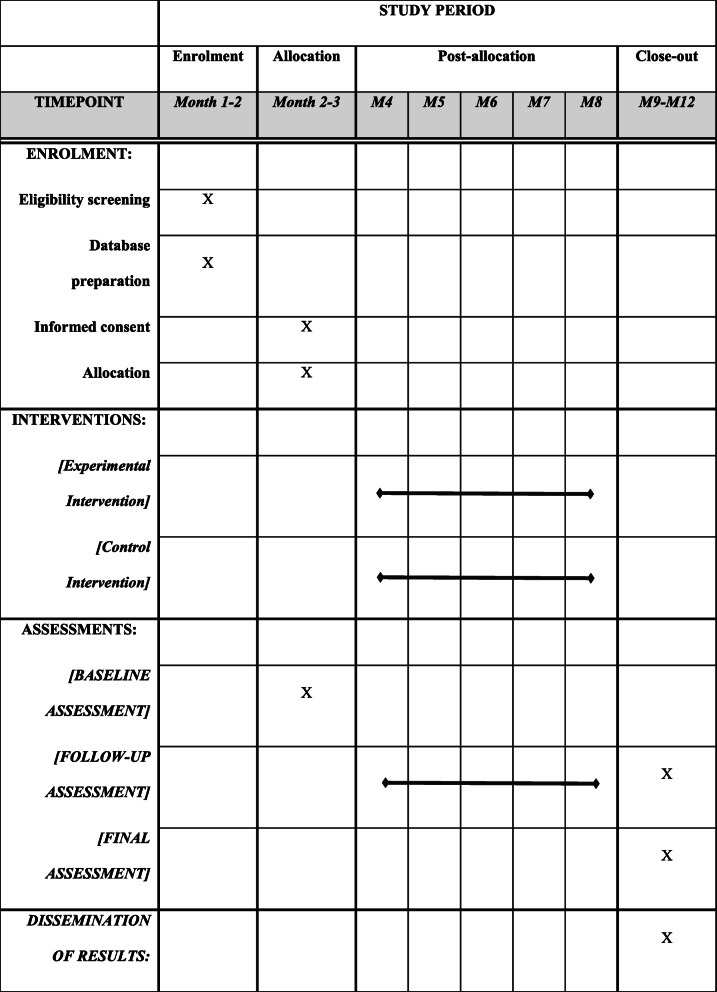
Timeline and dimensions assessed at the time points

#### Description of the variables

For the principal variable being manipulated, we will study dyspnoea (Medical Research Council, MRC) and the degree of dependence in carrying out the activities of daily living (Barthel Index, BI). And as secondary variables, we have the patient’s physical performance (via the Short Physical Performance Score, SPPB), their fear/avoidance of movement (Tampa Scale of Kinesiophobia–Fatigue, TSK-F), the oxygen saturation in the blood (pulse oximetry), and the quality of life of the oncology patient (ECOG performance status scale). Other intervening variables include those gathered from the patient’s clinical history, such as pathological diagnosis, number of lines of treatment, and socio-demographic and anthropometric data (sex, age, weight, height, and BMI).

#### Tools employed in the evaluation of the variables

1. “Barthel Index, (BI)” [18]: to evaluate dependence. Measures physical disability with proven validity and reliability. It is easy to apply and to interpret. Useful for evaluating the patient’s functional independence in the activities of daily living (ADL). Scored from 0 to 100, it quantifies the individual’s degree of dependence.
2. “Medical Research Council dyspnoea scale (MRC)” [19], to evaluate dyspnoea. The aim is to make it possible for the patients to quantitatively grade their own dyspnoea in a simple, visual manner. It establishes 5 degrees of dyspnoea when carrying out activities.
3. “Short Physical Performance Score (SPPB)” [20], to evaluate physical performance. The short physical performance battery, proven in our area for primary health care, is a test designed specifically to predict disabilities, and it has demonstrated the capacity to predict adverse events, dependence, institutionalisation, and mortality.
4. The “Tampa Scale of Kinesiophobia–Fatigue (TSK-F)” [8, 9], was developed to evaluate the fear of movement related to fatigue/pain. It has been proven effective in oncology patients and those with chronic fatigue syndrome. We will use the model with 11 items (TSK-F-11).
5. “Pulse oximetry” [21]: a non-invasive technique which uses a pulse oximeter to measure the saturation of oxygen (Sat O_2_) in the haemoglobin in circulating blood, usually in arterial blood.
6. “ECOG performance status scale” [22], is a practical way to measure the quality of life of cancer patients, whose life expectancies may change within a period of months, weeks, or even days. It was designed by the Eastern Cooperative Oncology Group (ECOG) in the United States and validated by the World Health Organization (WHO). The primary function of this scale is to objectify the patient’s quality of life or performance status.

The variables will be recorded on an individual sheet for each patient and stored afterwards in a database designed specifically for this study.

#### Interventions

The conventional standard of care will be maintained in both groups, as well as the lines of treatment prescribed by the Medical Oncology Service of the CAUSA. Additionally, all subjects in both groups will receive an individualised prescription of multimodal physical exercise.

In the experimental group, we will also implement an intervention with a programme for recovering the individual’s autonomy by means of reeducation in the activities of daily living.

The *oncological functional reeducation programme* will comprise the following actions:

1. Multimodal physical exercise programme: this therapeutic measure will be applied for all individuals, in both the experimental group and the control group. It will consist of two short sessions each day, one in the morning and another in the afternoon, of 15–20 min each. The dose and load of the programme, as well as any special considerations, will always be adapted to the functional capacities of each patient, in accordance with the assessments carried out. Additionally, we will always take into consideration the patient’s symptoms at the time, as there may be individual changes in a very short period of time. The directive will be to maintain a multimodal exercise programme to complete different exercises, including aerobic exercises, balance exercises, and low-load resistance training exercises targeting specific muscle groups, in both the upper quadrant and the lower quadrant. The sessions will be done following the recommendations of the American College of Sports Medicine (ACSM) [23], with an initial warm-up (2–3 min), a main part (8–12 min), and a final cool-down and relaxation (5 min)
  It will be permitted—and recommended—to do the exercises with the patient’s specific oxygen therapy range, if they have one. The patient’s oxygen saturation levels will also be measured before, during, and after completing the exercise, to ensure that there are no clinical alterations, which may contraindicate the therapeutic practice.
2. Reeducation in the activities of daily living: Intensity and simplification of the activities and trainings of energy-saving techniques (EST). An individual training will be completed on the rules for the simplification of activities, consisting of [24]:
  Exhaustive organisation of the workspaces where the activities are to be carried out
  Adaptation of the work plans and placement of the objects within the patient’s reach
  Prioritising basic self-care activities while sitting
  Movement control: doing the movements in a slow, coordinated, and harmonious manner, avoiding impulsive and vigorous movements
  Alternating heavy activities with light activities, establishing the required rest times between them
  Combining periods of balanced activity with periods of rest
  An individualised assessment will also be completed where we will prescribe the support products which will facilitate the patient’s mobilisation when completing their activities of daily living, such as a walker with an adaptation for oxygen therapy (a portable oxygen concentrator).
  And finally, we will complete an exhaustive daily log of the activity that the patient does, which we will in turn use to modify the same activity, adapting it to the patient’s clinical situation at all times. In this way, we will establish a proper pattern, which will lead the patient to improve their autonomy, as they will be using all of their capacities available at the time, without exceeding them or discounting them. This will be done using a diary which the patient will complete each day and which the investigator responsible for the intervention will supervise daily.

### Data analysis

The statistical analysis will be carried out with the intention to treat. In the descriptive analysis, the population is described by presenting the data as means and standard deviations for the continuous variables and as frequency distributions for the qualitative variables. To evaluate the comparability in the baseline assessment between the two study groups, we will employ the Chi-squared test for qualitative variables and the Student’s T-test for the comparison of the means between the two groups, in addition to the Kolmogorov–Smirnov test to find the normality of the sample. In the inferential analysis, we will consider a confidence interval of 95% (*p* < 0.05), and will use Student’s T-test for related pre and post samples and for independent samples at the end of the study, in addition to a multivariate analysis of variance (MANOVA) with repeated measures (3 levels) to assess interactions between categorical variables, and to determine significant differences between groups and within groups. Additionally, to assess the magnitude of the change in the variables, the effect size of the interventions will be calculated as Cohen’s d (small (0.2), medium (0.5), and large (0.8)) and the partial eta squared (ƞ2p) (small (0.01), medium (0.06), and large (0.14)), where significant, as appropriate with the statistical test applied [25]. We will use Pearson’s Correlation Coefficient to analyse the correlations between the various intervening variables and the parameters established in the different questionnaires and scales employed. A survival follow-up analysis will be completed using the Kaplan–Meier non-parametric method to learn the time of the intervention until the patient’s discharge from hospital, and the survival curves of each of the groups in the study will be compared using the non-parametric logrank test. Processing and analysing the data will be done using the statistics pack SPSS 25.0.

### Ethical considerations

The study will be carried out thanks to the authorisation of a Clinical Research Ethics Committee of the Area of Health of Salamanca (ID: PI 202007547), having obtained the prior written informed consent of the study subjects and in conformance with the Helsinki Declaration. In it, participants will be informed of the objectives of the project, as well as the risks and benefits of the interventions to be carried out, explained by one of the main investigators. The confidentiality of the subjects included will be guaranteed at all times, in conformance with the laws regarding the protection of personal data and biomedical investigation present in the articles of Spanish Organic Law 3/2018 of 5 December on the protection of personal data and the guarantee of digital rights, and in Regulation (EU) 2016/679 of the European Parliament and of the Council of 27 April 2016 on data protection (GDPR), and within the conditions established by Law 14/2007 on biomedical investigation.

Significant modifications to the protocol (such as changes in the tools of evaluation, modifications to the selection criteria or to the interventions) will be communicated immediately to the Ethics Committee.

And since this is a randomised clinical trial, it follows the CONSORT guidelines, and it was registered. TRIAL REGISTRATION: ClinicalTrials.gov NCT04766593.

### Rigour

This protocol study also follows the evidence-based recommendations of The SPIRIT 2013 Statement for the minimum content of a clinical trial protocol. And the design of the study also follows the evidence-based, minimum set of recommendations of the CONSORT 2010 Statement for conduct parallel-group randomized controlled trials (RCT), enabling readers to understand a trial’s design, conduct, analysis and interpretation, and to assess the validity of its results.

## Discussion

If it is true that there has been an exponential increase in the studies related to the implementation of physical exercise in the intervention of patients with respiratory pathologies, there still remains a problem with the generalisation that those individuals achieve that symptomatic improvement in the completion of their activities of daily living.

The present study will attempt to investigate the influence that rehabilitative measures that are oriented exclusively towards functional recovery and adaptation will have with respect to the measures of physical exercise employed with oncological patients with respiratory symptomatology.

In our daily clinical practice, we have observed first-hand the beneficial effects of physical exercise in the physical improvement of patients with dyspnoea. But we have also observed difficulties with the generalisation of this physical improvement in terms of occupational performance.

Based on the results obtained, we consider it indispensable to establish specific care protocols which improve not only the symptomatology, but also the individual in a more integral manner, which we believe will have positive repercussions on their quality of life and on that of the people around them. Dyspnoea is a symptom that is clearly incapacitating and which increases individuals’ dependence, which is then linked to an exceeding personal cost for their caretakers. By improving patients’ levels of dependence, we are contributing to the reduction of this problem for the caretakers.

### Limitations

The study follows all of the recommendations of CONSORT, but due to the nature of the intervention itself, the participating subjects will not be blinded. However, to minimise any contamination between groups, the investigator responsible for the study measurements at each assessment and the statistical analysis thereof, will be blinded.

### Dissemination plan

The investigator team is planning a rapid and broad diffusion of the results so as to guarantee the maximum visibility of this study. With this aim, the results of the study will be published in open-access, peer-reviewed scientific journals. We will have at least one publication of the primary results, and others planned with the secondary results. This will complement the presentation of the study results at important national and international scientific conferences and seminars. Similarly, we will do a diffusion on social media and other media.

### How potential changes in the study will be approached

Significant modifications to the protocol (such as change in the tools of evaluation, modifications to the selection criteria or to the interventions) will be communicated immediately to the bioethics committee.

## Conclusions

This manuscript presents the protocol of a study aimed at assessing the results of a model of care based on a comprehensive rehabilitation combining a usual multimodal physical exercise programme and a specific programme of recovery of autonomy through activities of daily living training, on functionality, respiratory parameters and quality of life in oncology patients with dyspnea.

## Data Availability

Upon completion of the study, the datasets generated and/or analysed during the current study will be available to those investigators who request it through FAIRsharing of the repositories of research data at https://www.re3data.org/.

## Abbreviations

MRC: Medical research council
SPPB: Short physical performance score
ECOG: Eastern Cooperative Oncology Group
ADL: Activities of daily living
MMSE: Mini-mental state examination
BI: Barthel index
TSK-F: Tampa Scale of Kinesiophobia–Fatigue
WHO: World Health Organization
ACSM: American College of Sports Medicine
EST: Energy-saving techniques
EU: European Union
